# A two-dimensional algebraic quantum liquid produced by an atomic simulator of the quantum Lifshitz model

**DOI:** 10.1038/ncomms9012

**Published:** 2015-08-13

**Authors:** Hoi Chun Po, Qi Zhou

**Affiliations:** 1 Department of Physics, The Chinese University of Hong Kong, Shatin, Hong Kong; 2 Department of Physics, University of California, Berkeley, California 94720, USA

## Abstract

Bosons have a natural instinct to condense at zero temperature. It is a long-standing challenge to create a high-dimensional quantum liquid that does not exhibit long-range order at the ground state, as either extreme experimental parameters or sophisticated designs of microscopic Hamiltonians are required for suppressing the condensation. Here we show that synthetic gauge fields for ultracold atoms, using either the Raman scheme or shaken lattices, provide physicists a simple and practical scheme to produce a two-dimensional algebraic quantum liquid at the ground state. This quantum liquid arises at a critical Lifshitz point, where a two-dimensional quartic dispersion emerges in the momentum space, and many fundamental properties of two-dimensional bosons are changed in its proximity. Such an ideal simulator of the quantum Lifshitz model allows experimentalists to directly visualize and explore the deconfinement transition of topological excitations, an intriguing phenomenon that is difficult to access in other systems.

## Abstract (web summary)

It is challenging to create a high-dimensional quantum liquid that does not exhibit long-range order at the ground state, as condensation is in practice unavoidable. Here the authors propose a scheme on how to use synthetic gauge fields to produce a two-dimensional algebraic quantum liquid at the ground state.

## Graphical abstract

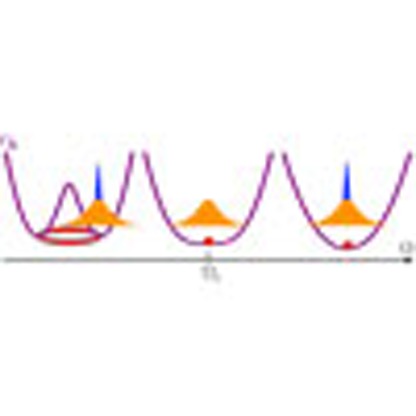

Bosons are well-known for preferring to form a Bose-Einstein condensate at low temperatures. Such is the case for most bosonic systems in three dimensions. In lower dimensions, the reduced coordination enhances quantum fluctuation, and Bose-Einstein condensate is either absent (one dimension) or confined to strictly zero temperature (two dimensions). Whereas these textbook results of the ground state of bosons are intrinsically determined by the fundamental Bose-Einstein statistics and can be qualitatively understood in the non-interacting limit, there have been intensive interests to explore schemes for suppressing condensation at zero temperature. The success of such an effort will pave the way for creating novel quantum many-body ground states without ordering^1^,^2^,^3^,^4^,^5^,^6^,^7^,^8^,^9^,^10^. However, a challenge is that the currently devised schemes require either extreme experimental conditions, such as a fast rotation of an atomic cloud at a frequency extremely close to that of the trapping potential, or delicate designs of sophisticated Hamiltonian, such as lattice models containing ring exchange or even more complicated terms. The lofty goal of experimentally realizing a non-condensed quantum liquid as the ground state in high dimensions has not yet been achieved.

Synthetic gauge field is one of the most important topics in current studies of ultracold atom physics. One scheme for realizing synthetic gauge fields is to use Raman beams to couple hyperfine spin states and the motion of the atoms. Using this scheme, a spin-orbit coupling (SOC) has been created for neutral atoms in laboratories^11^,^12^,^13^,^14^,^15^,^16^. The other scheme is lattice shaking, which produces gauge fields by dynamically driving the lattice itself without resorting to extra external fields^17^,^18^,^19^,^20^,^21^. The dynamically induced band hybridization can also be viewed as an effective SOC, where different bands represent different components of a pseudospin^22^.

While the current interest on SOC has been mainly focusing on topological matters, here we show that synthetic gauge fields, realized using either the Raman scheme or shaken lattices, provide one an unprecedented means to suppress the condensation in two dimensions even at zero temperature and produce an algebraic quantum liquid as the ground state of interacting bosons. The algebraic quantum liquid here is induced by a two-dimensional (2D) quartic dispersion at a Lifshitz point, where the quadratic term of the spatial gradient vanishes in the Hamiltonian describing low-energy physics. Surprisingly, this leads to a natural realization of quantum Lifshitz model, an important theoretical tool for studying a wide range of exotic phenomena in modern physics, including the deconfinement transition in condensed matter physics^23^,^24^,^25^,^26^,^27^,^28^ and quantum gravity in high-energy physics^29^,^30^,^31^,^32^. Despite its profound applications in fundamental physics, such a model has not been realized in a realistic system before. This is due to the difficulty in suppressing the quadratic term of the spatial gradient, which is always dominant in the low-energy effective theories for ordinary quantum many-body systems. Here we show that the high controllability of ultracold atoms allows one to create an ideal simulator of quantum Lifshitz model, which leads to a controllable scheme to access the Lifshitz point where exotic quantum phenomena occur. This atomic simulator can be used to directly probe the intriguing phenomena, including vanishing Berezinskii–Kosterlitz–Thouless (BKT) transition temperature and the deconfinement transition of vortices in superfluids.

## Results

### Quartic dispersion and effective dimension reduction

We consider a general Hamiltonian for 2D bosons with SOC that may be produced by the Raman scheme,


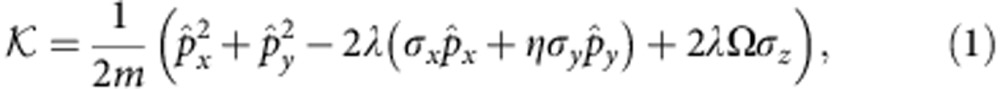


where *m* is the mass, 
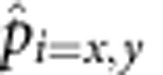
 is the momentum operator, *λ* and 0≤*η*≤1 characterize the strength and anisotropy of SOC, repsectively. *ℏ* and *k*_B_ are set to 1 in this study. For *η*=0, 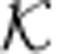 describes the synthetic SOC produced by the Raman scheme, where Ω is proportional to Raman frequency. For *η*=1, 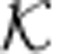 is identical to Rashba coupling with a magnetic field along the *z* axis. Many theoretical studies have proposed how to produce a SOC with finite strengths along multiple spatial directions^33^,^34^,^35^. Although a fully controllable Hamiltonian in equation (1) has not been realized in laboratories so far, it is very desirable to perform a systematic theoretical study on such a general model and to explore the novel phenomena it engenders.

**Figure 1:**
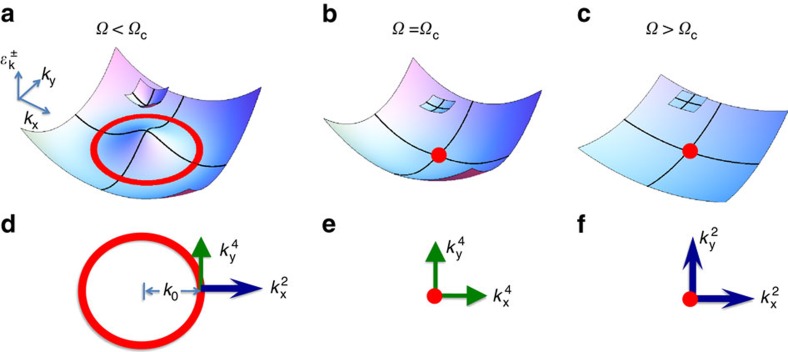
Schematics of single-particle dispersions at different Raman frequencies. (**a**) The minimum of 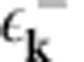 forms a circle if Ω<Ω_c_. 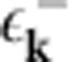 is quadratic and quartic along the radial and tangent direction of the circle, respectively. The plots are coloured only to help visualization. (**b**) The circle shrinks to a single point at **k**=0 when Ω=Ω_c_. At this critical point, 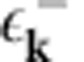 becomes quartic at small |**k**|. (**c**) If Ω>Ω_c_, the minimum remains to be a single point in the momentum space, and 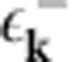 becomes quadratic again at small |**k**|. (**d**–**f**) Top view of the single-particle energy minimum, respectively, for (**a**–**c**). Blue long and green short arrows represent the quadratic and quartic dispersions along the *k*_*x*_ and *k*_*y*_ directions.

We start from the isotropic limit where *η*=1. For fermions, this model has been extensively studied in the context of topological matters. For bosons, it has been much less explored except for the special case with Ω=0. Including a finite Ω, the kinetic energy can be written as


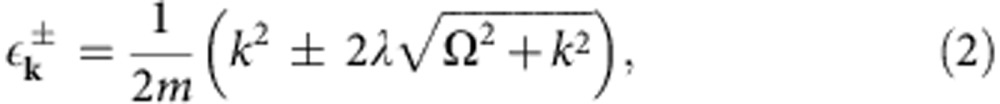


where ± corresponds to the upper and lower branch, respectively, and *k*=|**k**|. It is straightforward to show that there exists a critical value 
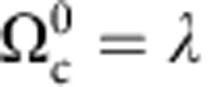
. If 
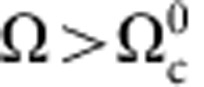
, 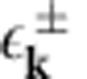 has a unique minimum at **k**=**0**. When 
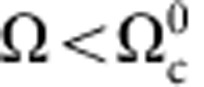
, the minima for 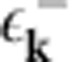 forms an infinitely degenerate circle with a radius 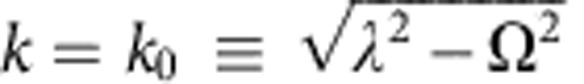. This circle shrinks to a single point at **k**=**0** when 
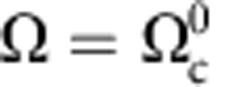
, and 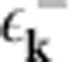 at small *k* may be expanded as


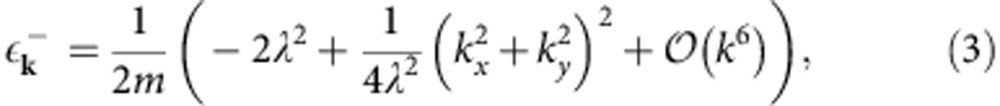


which becomes quartic instead of the conventional quadratic ones for ordinary particles, as shown in Fig. 1. If one considers the identity, 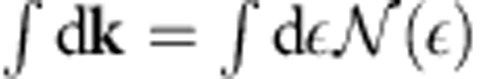, where 
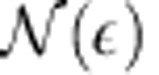
 is the density of states and 
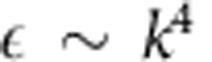
 at small *k*, one immediately sees that 
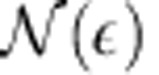
 at low energies 
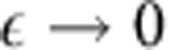
 becomes 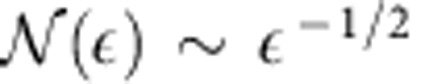, similar to the one of an ordinary one-dimensional system. Without physically reducing the dimension of the system, for instance, by imposing a strong confinement potential to completely quench the kinetics along one spatial direction, SOC here partially suppresses the kinetic energy along all spatial directions through changing the ordinary quadratic dispersion to a much more flattened one ∼*k*^4^, and leads to an effective dimension reduction. As seen from the density of states, such an effective dimension reduction allows one to directly conclude that for non-interacting bosons, a condensate is absent even at zero temperature when 
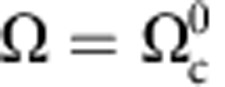
. If one includes interaction effects, as shown later, such a fully quartic dispersion along all the spatial directions is the microscopic origin for the rise of a Lifshitz point in the low-energy effective theory^36^,^37^.

Interestingly, a similar quartic dispersion in two dimensions can be produced in a shaken square lattice with four-fold rotation symmetry. The potential of such a lattice can be written as 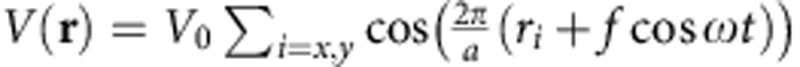, where *ω* and *f* are the shaking frequency and amplitude, respectively. In such a separable lattice, the band gap *E*_g_ between the *s* and *p* bands is identical to that between the *d*_*xy*_ and *p* bands. When *ω* is tuned to be close to the band gap *E*_g_, these four bands are coupled by the photon-assisted hybridization. Within the rotating wave approximation, the momentum-space single-particle Hamiltonian can be written as


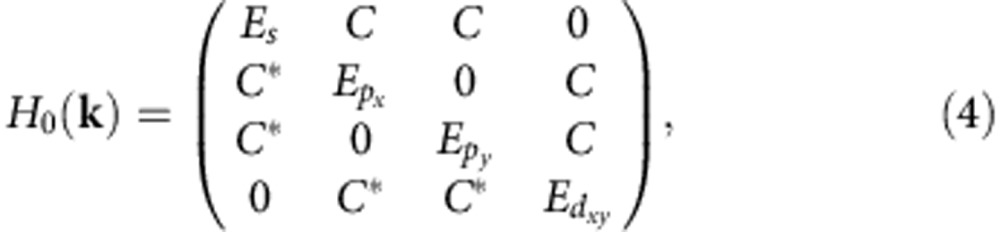


where *E*_*s*_=−*t*_*s*_(cos(*k*_*x*_*a*)+cos(*k*_*y*_*a*))+δ^L^, 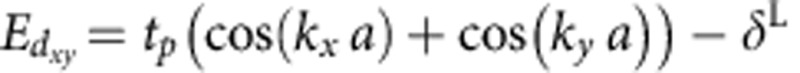, 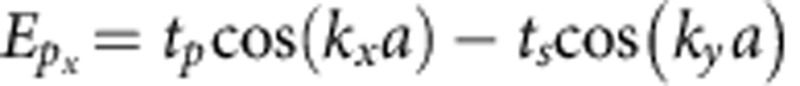, 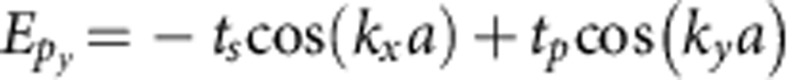, *t*_*s*_/2 and *t*_*p*_/2 are the tunnelling amplitudes, δ^L^=*ω*−*E*_g_ is the detuning, and the interband coupling *C* is controlled by *f* (Supplementary Note 1). *E*_*s*_ and 
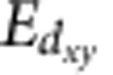
 are the energies of the side bands formed by the *s* and *d*_*xy*_ orbitals through absorbing and emitting one photon, respectively. At small *k*, the energy of the dressed *s* band can be expanded as


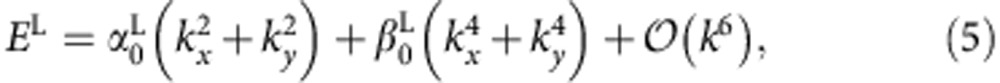


where 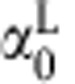 has a simple expression in the limit *C*≪δ^L^,


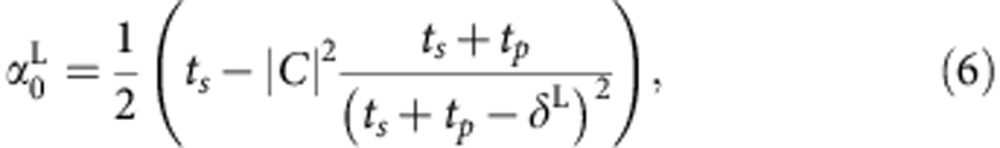


and the expression for 
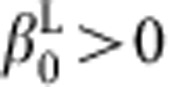
 is given in Supplementary Note 1.

From equation (6), one observes that tuning *f* or δ^L^ could suppress 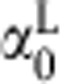, similar to tuning Ω in the Raman scheme(Supplementary Fig. 1). For instance, there exists a critical value 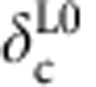, where the energy of one band can be written as


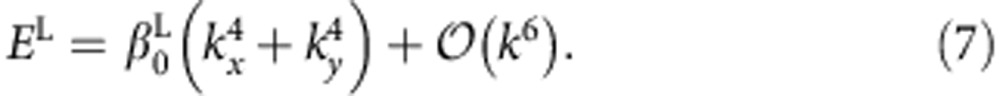


Despite the term 
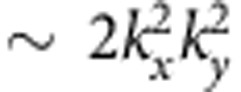
 is absent in equation (7), the low-energy density of states can be still written as 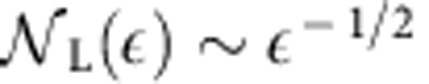. One therefore observes that lattice shaking could also suppress the condensation at the ground state in two dimensions. Similar to the Raman scheme, there is a unique energy minimum at **k**=**0** when 
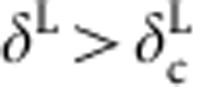
 and four energy minima, related by four-fold rotation symmetry, when 
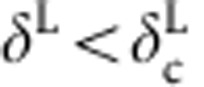
. As the system is tuned from the zero- to the finite-momentum phase across a continuous phase transition, 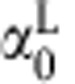 will necessarily change sign and hence 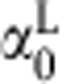 also vanishes at the critical point.

### A simulator of quantum Lifshitz model

We first consider interaction effects in the Raman scheme. Ultracold bosons interact through a contact potential,


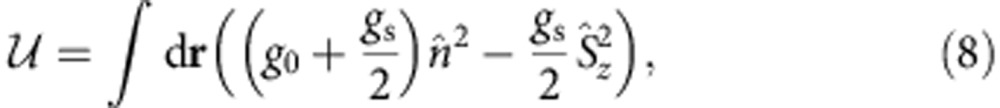


where 
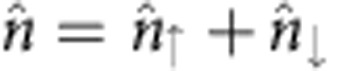
 is the total density operator, and 
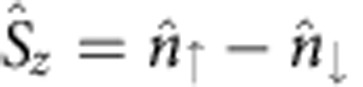
. 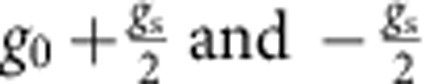 characterize the strength of density-density interaction and spin-dependent interaction respectively. Mean field solutions are pursued by minimizing the ground state energy using the ansatz


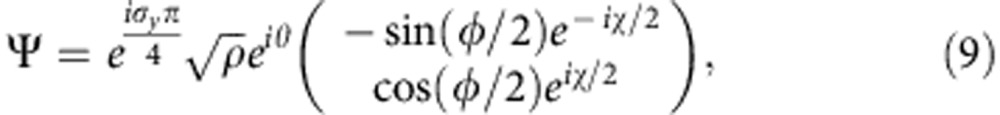


where *ρ* and *θ* are the density and the phase, respectively. *φ* and *χ* characterize the spin orientation. A unitary transformation 
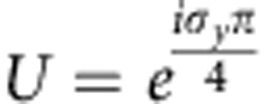
 is introduced to avoid the ambiguity in defining χ at the north and south poles of the Bloch sphere.

Mean field results show that interaction shifts the critical point to Ω_c_=*λ*−*g*_s_*mρ*/*λ*. Ω_c_ reduces to 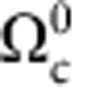 if *g*_s_=0. The mean field value of the phase *θ*_0_ is 0 and 
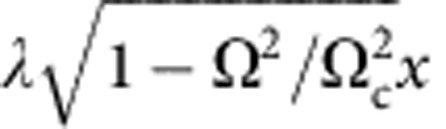
 for Ω>Ω_c_ and Ω<Ω_c_, respectively, analogous to the zero-momentum^38^ and plane-wave condensate^39^,^40^ in three dimensions. While both states break *U*(1) symmetry, the latter one also breaks the rotation symmetry in momentum space, as interaction lifts the infinite degeneracy on the circle of kinetic energy minimum^41^. Another (first-order) transition from the plane-wave phase to a stripe phase at 
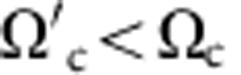
 is not relevant to our discussions here. To include quantum fluctuations, we introduce *ρ*=*ρ*_0_+δ*ρ*, *θ*=*θ*_0_+δ*θ*, *χ*=*χ*_0_+δ*χ* and *φ*=*φ*_0_+δ*φ*, where the subscript 0 denotes the mean field values. δ*ρ*, δ*χ* and δ*φ*, which are massive due to repulsive interaction and spin-momentum locking induced by SOC, respectively, are integrated out. The gapless phase fluctuation δ*θ* is incorporated using imaginary time path integral, 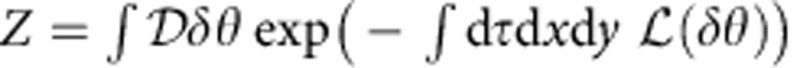, where *τ*=*it*, and 
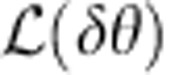
 is an effective low-energy Lagrangian. To simplify notation, we relabel δ*θ* as *θ* in the following discussions.

For Ω≥Ω_c_, we obtain


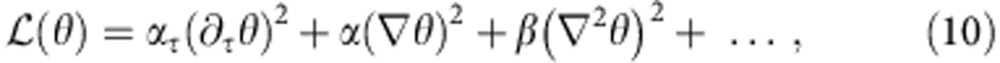


where 
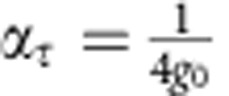
, 
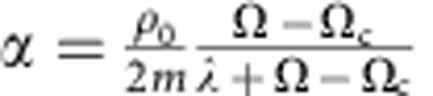
, 
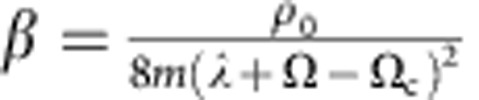
, and the ellipsis represents terms that are higher order in derivative expansion or contribute to observables only as higher order corrections (Supplementary Note 2). It is interesting to note that equation (10) describes the Quantum Lifshitz model, an important tool in both condensed matter and high-energy physics^23^,^24^,^25^,^26^,^27^,^28^. Whereas such a model has not been realized in a realistic system before, synthetic SOC naturally provides physicists an ideal simulator of it, since all parameters in equation (10) are well controlled. In particular, it allows one to access the Lifshitz point, where *α*(∇*θ*)^2^ vanishes, by tuning Ω to Ω_c_. A sequence of exotic phenomena emerges here, such as the suppression of condensation, the rise of an algebraic bosonic liquid and the deconfinement of topological excitations. Unlike other systems where the Quantum Lifshitz model remains a purely theoretical description, the field *θ* here directly corresponds to physical observables of ultracold atoms, and all the above intriguing phenomena can be experimentally probed.

### Suppressed condensation and algebraic quantum liquid

Condensate density is provided by *n*_0↑_=0 and 
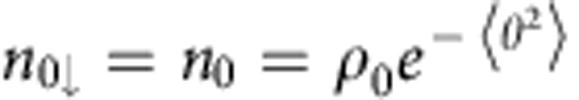
. As 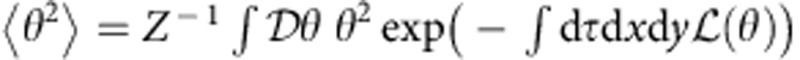, we have


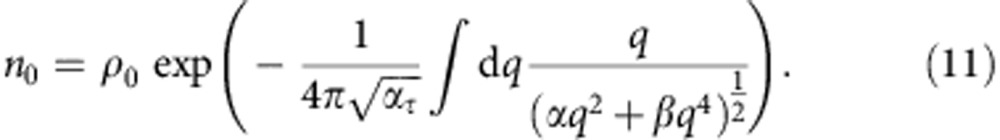


When *α*=0, the sound velocity vanishes and the low-lying excitation spectrum becomes 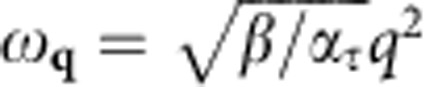. Such an unconventional collective excitation spectrum fundamentally changes thermodynamic properties of the system. For instance, it leads to a linear-specific heat at low temperatures


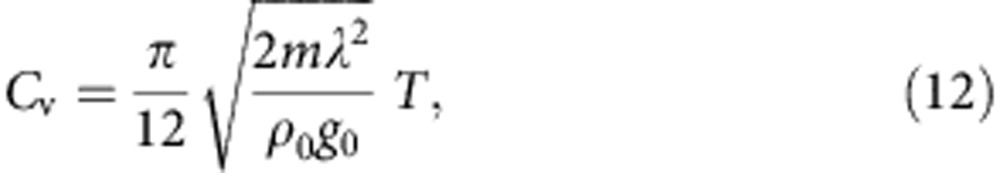


different from the conventional *T*^2^ behavior in ordinary 2D bosons. More importantly, one notes an infrared divergence ∫d*q q*^−1^ in equation (11), which usually occurs in one dimension for ground states of ordinary bosons. Such a divergence here destroys the 2D condensation of interacting bosons at *T*=0. This is a purely quantum effect, different from thermal fluctuation suppressed condensation in either two or three dimensions^42^,^43^. The characteristic long-range order at the ground state of ordinary 2D systems is then replaced by an algebraic one. The one-body correlation function 
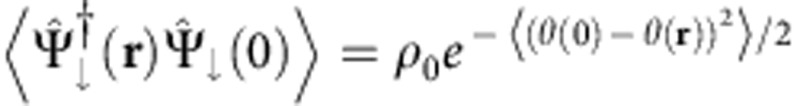
 becomes power-law like,


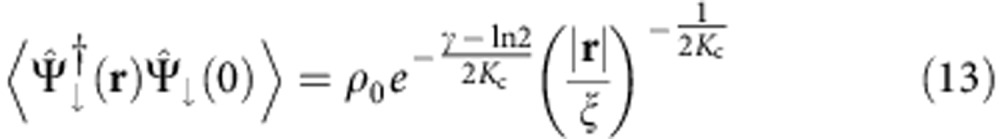


where *γ* is the Euler-Mascheroni constant, and *ξ*=(2*mρ*_0_*g*_0_)^−1/2^ is the healing length. 
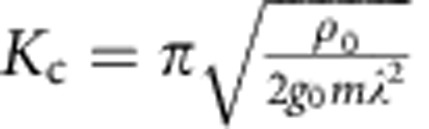
 is an effective Luttinger liquid parameter characterizing this algebraic quantum liquid.

While we focus on infinite systems in this study, it is worth mentioning how the finite size of a system affects the physics at the Liftshitz point. In a finite system with linear size *R*, the momentum-space integrals are regularized in the infrared by *R*^−1^, and hence the infrared divergence in the integral in equation (11) is removed even when *α*=0. As with an ordinary one-dimensional finite system, this produces a finite-size effect induced condensation with condensate fraction


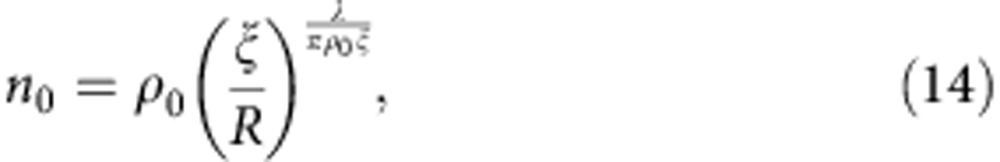


which shows that *n*_0_ decreases as a power-law function of the linear size of the system and eventually vanishes in the thermodynamic limit.

Near the critical point, although *n*_0_ becomes finite, its amplitude is strongly suppressed due to the smallness of *α*. We define q* by αq^*2^=βq^*4^. As the quadratic and quartic terms are relatively more important in the Lagrangian for *q*<q* and *q*>q*, respectively, the integral in equation (11) may be approximated by 
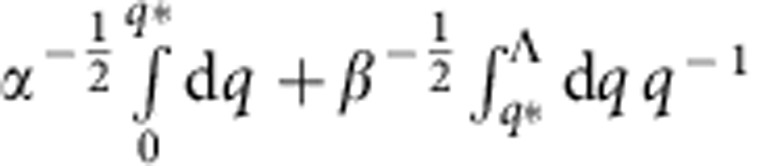
, where Λ=*ξ*^−1^ is a large momentum cutoff. Within this approximation, we obtain the scaling form for the condensate density near the critical point,


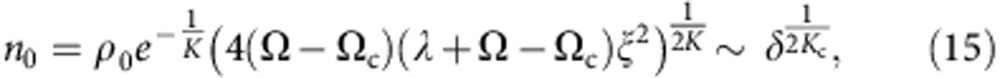


where *K*=*λK*_c_/(*λ*+Ω−Ω_c_) and δ=(Ω−Ω_c_)/Ω_c_>0. This result shows that synthetic SOC provides one a unique tool to control the condensation at the ground state without sophisticated designs of the microscopic Hamiltonian. equation (15) is verified by an numerically evaluating equation (11).

When Ω<Ω_c_, *α* becomes negative, and the phase gradient becomes finite. The low energy effective Lagrangian is reformulated around the new class of mean field solutions, and we obtain


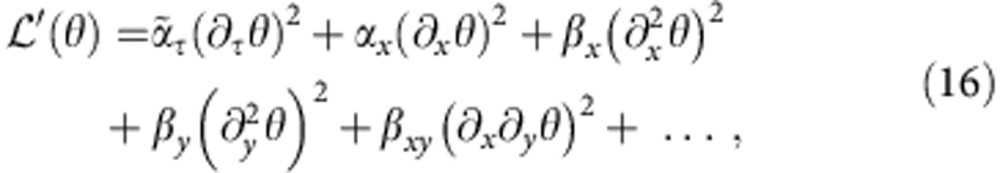


where the lengthy expressions for the coefficients are given in the Supplementary Note 2. When Ω=Ω_c_, 
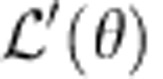
 and 
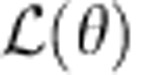
 become identical. If Ω<Ω_c_, 
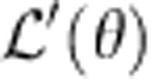
 has only one quartic mode along the *y* direction. This feature is inherited from the single-particle picture, in which 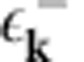 has an infinite degeneracy on the circle *k*=*k*_0_, similar to the case with Ω=0 (refs 42, 44). A single quartic mode in two dimensions is not sufficient to destroy the long-range order at *T*=0, and the condensate fraction becomes finite again when Ω decreases from Ω_c_, that is, 
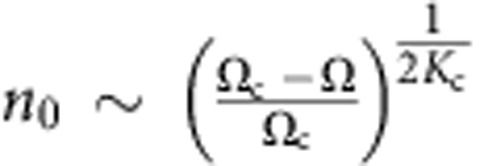
.

**Figure 2:**
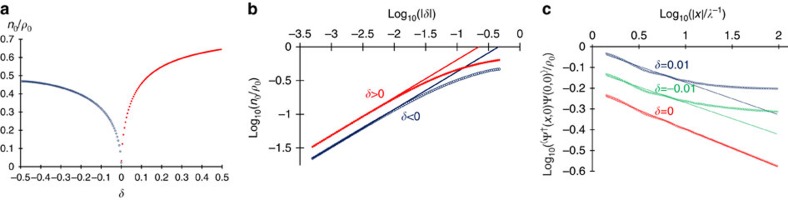
Condensate fraction and correlation function. (**a**) Condensate fraction as a function of δ=(Ω−Ω_c_)/Ω_c_ evaluated using parameters *ρ*_0_=0.125*λ*^2^, *g*_0_=0.8*m*^−1^, *g*=−0.001*g*_0_. At the critical point δ=0, condensate fraction is suppressed down to zero. Near the critical point, it scales as a power-law function of δ on both sides of the critical point. (**b**) Log–log plot of **a**, which directly shows the power-law scaling when δ→0. (**c**) Log–log plot of correlation function at different δ evaluated using parameters *ρ*_0_=1.25*λ*^2^, *g*_0_=0.8*m*^−1^, *g*=−0.001*g*_0_. At the critical point, the correlation function is completely a power-law function. The linear fit provides *K*_c_=2.72(1), close to the analytical result 2.78. Near the critical point, the correlation function approaches a constant when |*x*|→∞, and the power-law feature emerges in an intermediate length scale. The curves in the log-log plots are vertically offset for clarity.

The algebraic bosonic liquid at Ω=Ω_c_ and the strong suppression of condensation near this Lifshitz point can be directly probed by measuring the single-particle correlation function in the real space using a Ramsey-like method, in which the *in situ* inteference fringes of two copies of the bosonic sample is measured^45^. It can also be extracted from the measurement of the momentum distribution^46^. When Ω=Ω_c_, 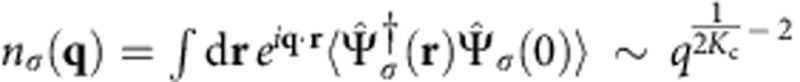. Such a power-law singularity signifies the algebraic order. In the vicinity of Ω_c_, the power-law like feature readily emerges in an intermediate momentum scale q*<*q*<*ξ*^−1^, in which the quartic term in equation (10) dominates. This can be easily understood from the real-space correlation function. In the region *ξ*<|**r**|<1/q*, 
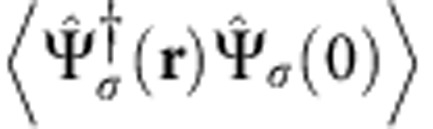
 is a power-law function, and approaches a constant *n*_0*σ*_ when |**r**|≫1/q*, as shown by the numerical results in Fig. 2. At the critical point, 1/q* diverges and 
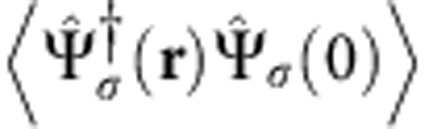
 remains algebraic at arbitrarily large distances. Expressions for the correlation function at different length scales are given in the Supplementary Note 2.

In typical ultracold atom experiments, a harmonic trap is present and it is useful to discuss the impact of the inhomogeneity on the single-particle correlation function discussed in this study. It is known that for weakly interacting bosons, the phase fluctuations dominate the low-energy physics even in a trap. The single-particle correlation function can be written as 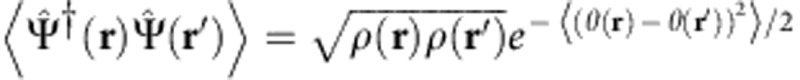, where *ρ*(**r**) is the density distribution in the trap^47^. By measuring *ρ*(**r**), the distortion of the single-particle correlation function due to the inhomogenous density distribution in the harmonic trap can be singled out, and the phase fluctuation in a large-enough trap is known to remain qualitatively the same as that of a homogenous system.

The inhomogeneity issue can even be eliminated in a flat box potential, which has been realized in recent experiments^48^. Such a box potential has the unique advantage of allowing a direct comparison between experimental results and theoretical calculations, since the latter are usually carried out for homogenous systems. In particular, a much more accurate momentum distribution can be obtained in the Time-Of-Flight experiment, since averaging over an inhomogeneous harmonic trap is no longer necessary. From the details of the momentum distribution, one can distinguish between different quantum many-body phases that exhibit different correlation functions, such as the long range, algebraic or the exponential order.

### Deconfinement transition and vanishing *T*_BKT_

We now turn to the deconfinement transition. For conventional 2D bosons, vortices are the characteristic topological excitations and are confined by a logarithmic force *U*_v_=2*πρ*_s_∫d**r**d**r**′*m*(**r**)ln|(**r**−**r**′)|*m*(**r***′*), analogous to the Coulomb force in 2D electrons^49^. *m*(**r**) is the density of vortices at **r** and *ρ*_s_ is the superfluid stiffness. A direct consequence of the confinement is a finite BKT transition temperature *T*_BKT_, below which free vortices are prohibited due to the binding of vortex and anti-vortex^50^. Synthetic SOC changes this fundamental property of 2D bosons.

At finite temperatures, the low-energy physics is dominated by the zero frequency mode *ω*=0 of the Lagrangian in equation (10). The effective Hamiltonian at *T*≠0 can be formulated as


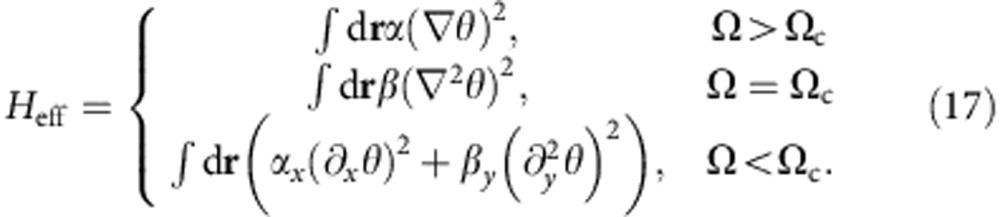


For Ω>Ω_c_, the Hamiltonian corresponds to an ordinary *XY* model. *ρ*_s_=*α*/2 is controlled by δ=(Ω−Ω_c_)/Ω_c_. When *α* decreases down to zero, *ρ*_s_ becomes zero, and the long range Coulomb interaction among vortices disappears. As ∇^2^*θ*=*m*(**r**), one obtains *H*_eff_=∫d**r***β*(∇^2^*θ*)^2^=*β* ∫d**r**d**r**′*m*(**r**)δ^2^(**r**−**r**′)*m*(**r**′) at Ω=Ω_c_, that is, the interaction between vortices becomes a short-range one^25^. Once a vortex and anti-vortex pair is created by thermal excitations, the short-range interaction could not prevent them from deconfinement. A direct consequence is then a vanishing *T*_BKT_. Near the critical point, *T*_BKT_ is given by the ordinary BKT theory,


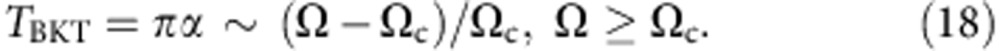


The vanishing *T*_BKT_ at the critical point can also be understood from a simple consideration of the free energy cost *F*_v_=*E*_v_−*TS*_v_ for creating a single free vortex excitation. In this picture *T*_BKT_ is the characteristic energy scale at which the vortex energy cost is balanced by its entropy gain. Such a balancing is possible in a thermodynamically large system only if *E*_v_ has the same logarithmic scaling in system size as does *S*_v_. At the critical point where *α*=0, 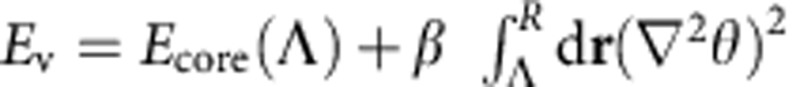, where Λ is a short-range cutoff proportional to the linear size of the core and *R* is the linear size of the system. Outside of the core region, since ∇^2^*θ*=0, the last term vanishes and *E*_v_ does not grow as *R* increases, unlike an ordinary vortex. Therefore, *TS*_v_ always dominates and vortices become free at any finite temperatures, that is, *T*_BKT_=0.

For Ω<Ω_c_, the Hamiltonian can be regarded as an extreme case of the anisotropic *XY* model


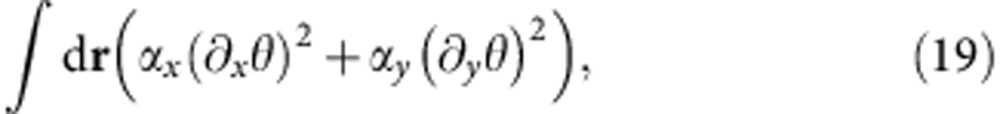


with *α*_*y*_=0. For *α*_*y*_>0, one could perform a simple rescaling along the *y* direction, and define 
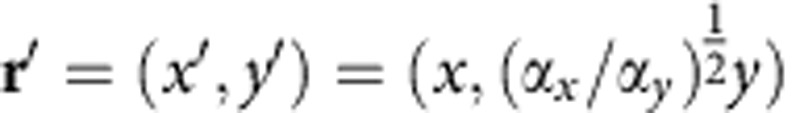
 so that 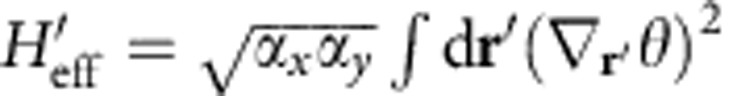. This gives 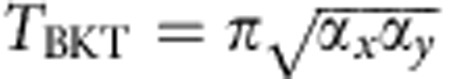, and hence *T*_BKT_ vanishes as *α*_*y*_→0, similar to the special case of Ω=0 studied before^44^. 
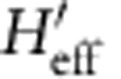
 shows that the logarithmic interaction between vortices vanishes if *α*_*y*_=0, and vortices are also deconfined, as a consequence of the vanishing sound velocity along the *y* direction.

*T*_BKT_ can also be calculated for anisotropic SOC, where 0≤*η*<1 and the Hamiltonian is an anisotropic XY model as in equation (19). The coefficients of the effective Lagrangian are provided in the Supplementary Note 2. There always exists a critical point Ω_c_(*η*), at which the coefficient of (∂_*x*_*θ*)^2^ vanishes. Due to the presence of (∂_*y*_*θ*)^2^ for 0≤*η*<1, the condensate fraction is finite and has a minimum at Ω_c_(*η*). This fact can be qualitatively understood in the non-interacting limit, where the single-particle spectrum for small *k* becomes


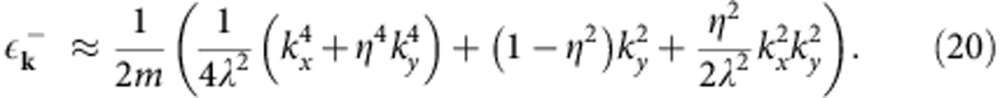


**Figure 3:**
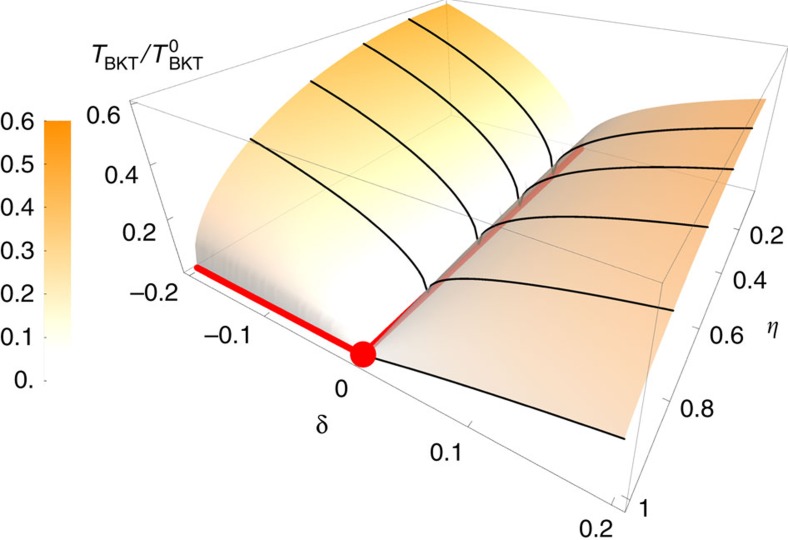
Suppression of BKT transition temperature. δ and η respectively parameterize deviation from Ω_c_ and the anisotropy of SOC. 
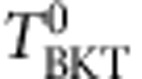
 is the BKT transition temperature in the absence of SOC. Black curves represent *T*_BKT_ at different fixed values of η. Red lines represent where *T*_BKT_ vanishes. For any values of *η*<1, *T*_BKT_ is suppressed down to zero at one critical point δ_c_(*η*). For the isotropic case *η*=1, *T*_BKT_ remains zero if δ≤δ_c_(*η*=1). The red dot indicates the Lifshitz point.

At Ω_c_(*η*), the density of states becomes 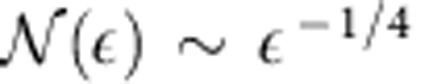, which does not lead to divergent quantum depletion at zero temperature. In contrast, *T*_BKT_ is still suppressed down to zero at Ω_c_(*η*) due to the vanishing (∂_*x*_*θ*)^2^ term in the Hamiltonian, as shown in Fig. 3, and vortices are deconfined at this point. As mentioned before, the current Raman scheme corresponds to the extreme case where *η*=0. Experimentalists are readily able to observe the quenched *T*_BKT_ in two dimensions.

By taking images of atomic densities, the distribution of vortices, including their locations and separations, have been measured^51^,^52^. Moreover, *T*_BKT_ has been measured using a variety of schemes^45^,^53^,^54^. This allows a direct visualization of the deconfinement transition in the system, and a vanishing *T*_BKT_ serves as a signature of such deconfinement. It is worth mentioning that near the Lifshitz point, *T*_BKT_ is strongly suppressed, and both the quantum and thermal fluctuations are important. Similar to other quantum critical phenomena, the scaling form of *T*_BKT_ as shown in equation (18) allows experimentalists to locate the deconfinement transition point, without tuning Ω exactly at Ω_c_, using finite temperature measurement. For typical current experiments, SOC energy scale (*λ*^2^/*m*) dominates over that of the spin-dependent interaction (*g*_s_*ρ*_0_)^11^, and one has 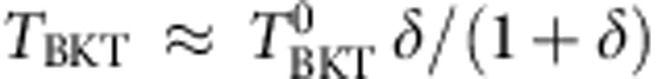, where 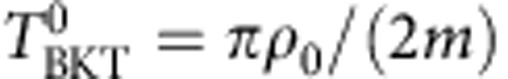 is the BKT transition temperature for an ordinary 2D Bose gas. Since 
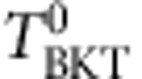
 is typically a few hundred *nK* (refs 45, 53, 54), the suppression of *T*_BKT_ can be readily observed in the currently available temperature scale. In addition, the scaling regime *T*_BKT_∼δ, which is valid when δ≪1, can be reached when the temperature is about a few or a few tens of *nK*. The proliferation of vortices and other intriguing phenomena in the proximity of the Lifshitz point can therefore be observed in such temperature regime by experimentally varying δ, which can be precisely controlled by tuning the Raman frequency.

## Discussion

We have presented a systematic analysis of the Raman scheme generated SOC for a wide range of Raman frequencies (Ω) and SOC anisotropy (η). A key observation is that the number of quartic modes in the low-energy effective theory determines the condensate fraction at the ground state and the BKT transition temperature *T*_BKT_. While one single quartic mode is sufficient to suppress *T*_BKT_ down to zero, two quartic modes are required to destroy the condensate at *T*=0. All these discussions can be directly generalized to shaken lattices, since the microscopic origins of the quartic modes do not affect the low-energy physics. For non-interacting particles, we have seen that one can also suppress condensation through lattice shaking. Although the rotation symmetry is lowered into a discrete one in a shaken lattice, the long-wavelength behavior of the system is qualitatively unchanged. This can be intuitively understood by noting that in effective Lagrangians like equation (10), the symmetry constraint on the quadratic piece *α*(∇*θ*)^2^, which governs the leading order scaling behavior of physical quantities, is identical for four-fold and continuous rotation symmetry (as long as the symmetry is unbroken). Hence, the qualitative behaviours of physical quantities in the zero-momentum phase and at the critical point are unaffected by the reduction to four-fold rotation symmetry, unless they depend crucially on the anisotropy of the quartic terms. Both the condensate fraction and *T*_BKT_ are insensitive to such detail. In the finite-momentum phase, however, the reduction in rotation symmetry is already manifest in the leading quadratic order, as we will discuss below.

More quantitatively, we analyse the shaken lattice model in equation (4), choosing the ansatz


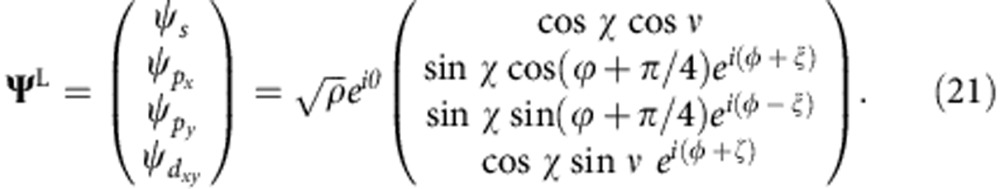


where *ρ* represent the total particle number density, and the angles *χ*, *ν* and 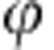 parameterize the relative particle numbers in the different bands. *θ*, *φ*, *ξ* and *ζ* parametrize the four independent phases of **Ψ**^L^. We carry out a thorough microscopic calculation, taking into account the inter- and intra-band interactions in lattices and integrating out massive modes, including the fluctuations of the densities and relative phases, and the results are presented in Supplementary Note 3. At a critical value 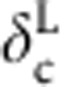, the low energy effective Lagrangian can be formulated as


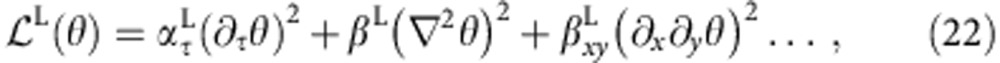


where the superscript L represents the results for shaken lattices. In particular, the microscopic calculation show that 
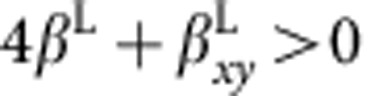
, which indicates the system is stable. The only difference with the Raman scheme is the last term in equation (22), which reflects the underlying discrete four-fold symmetry. While such a term modifies the Quantum Lifshitz model, it does not change all qualitative results discussed before. For instance, the condensate fraction at the critical point is given by


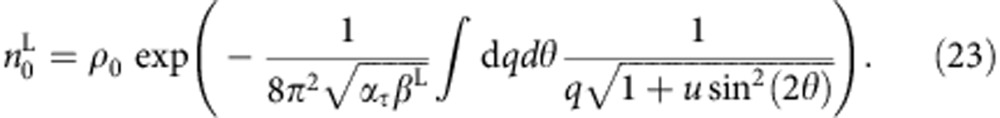


where 
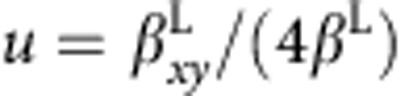
. While equation (23) reduces to equation (11) (with *α*=0 at Ω=Ω_c_) when *u*=0, the integral over *θ* for any finite *u*>−1 does not affect the infrared divergence. The condensate fraction therefore vanishes. Moreover, the correlation function decays algebraically, similar to the Raman scheme where 
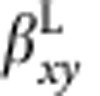
 is absent (Supplementary Figs 2 and 3). The conclusion *T*_BKT_=0 also remains unchanged. The energy of a single vortex becomes 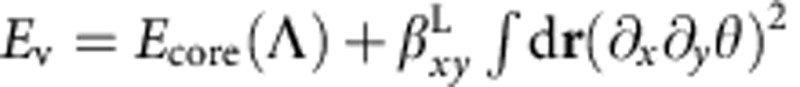. The last term can be written as 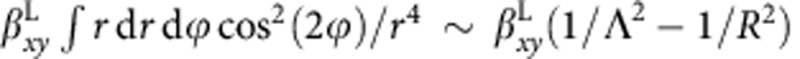, where 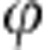 is the azimuth angle. The term depending on *R* decreases as *R* grows but entropy still grows logarithmically, and so in the thermodynamic limit *TS*_v_∼*T* ln(*R*/Λ) dominates over *E*_v_ at any finite temperatures, the same as the case for 
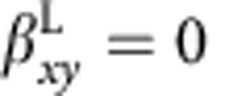
.

Away from the critical point 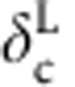, the quadratic terms dominate in the low-energy Lagrangian 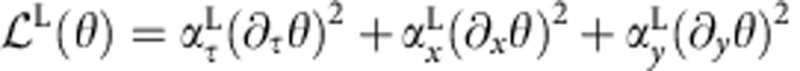. Unlike the isotropic Raman scheme with *η*=1 in the continuum, where a quartic mode still survive in the finite-momentum phase as shown in equation (16), the underlying lattice potential breaks continuous rotation symmetry and both 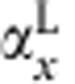 and 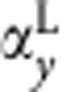 are finite away from 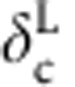. As a consequence, both the condensate fraction at the ground state and *T*_BKT_ increase from zero on both sides of the transition.

Another important feature of shaken lattices is that the shaking itself can be made anisotropic, that is, different shaking amplitude along the *x* and *y* directions. If *f*_*x*_≠*f*_*y*_, the quartic dispersions along the *x* and *y* directions cannot be simultaneously achieved, and there will be at most one quartic mode in the system, similar to the anisotropic Raman scheme with *η*≠1. The difference in shaking amplitude, δ*f*=*f*_*x*_−*f*_*y*_, plays the role of the anisotropy parameter *η* in the Raman scheme. When δ*f*≠0, the condensate fraction remains finite at *T*=0 but *T*_BKT_ still vanishes whenever there is a quartic mode emerging along any direction.

It is useful to mention possible heating effects in shaken lattices. In the current experiments^20^, there is only a loose confinement due to the harmonic trap along the transverse direction so that the 2D pancake could compensate possible heating effects in this one-dimensional shaken lattice. If the transverse direction is also tightly confined, a quantitative analysis is absent so far. A recent theoretical study indicates that by engineering the band structure, one should be able to suppress the two-body inter-band scattering. This reduces the undesirable occupation of the higher bands and hence the heating due to the decay can be, at least partially, suppressed^55^. Since it is unclear at this stage whether any other mechanisms, such as collective excitations induced by shaking, may also lead to considerable heating, a systematic study of the heating effect is desirable.

The study on 2D bosons has been a long-term important effort in the field of ultracold atom physics^45^,^53^,^54^. Although the presence of a condensate as the ground state and a finite BKT transition temperature have been familiar to physicists, we have shown that a synthetic SOC offers a simple and practical scheme to defeat these standard textbook results by suppressing the typical quadratic dispersion. Moreover, it leads to the realization of an ideal simulator of the quantum Lifshitz model for accessing intriguing phenomena that are important in many other fields, such as deconfinement transitions of topological excitations. As it is of fundamental interest in condensed matter physics community to explore quantum phases without ordering at the ground state, we hope that our work will stimulate more studies on using the highly controllable synthetic SOC for taming the ordering in many-body states of ultracold atoms and for exploring novel quantum phenomena that are not accessible in solids. We also hope that this atomic simulator of the quantum Lifshitz model may be useful for high-energy physics community on the topic of quantum gravity in the future.

## Additional information

**How to cite this article:** Po, H. C. & Zhou, Q. A two-dimensional algebraic quantum liquid produced by an atomic simulator of the quantum Lifshitz model. *Nat. Commun.* 6:8012 doi: 10.1038/ncomms9012 (2015).

## Supplementary Material

Supplementary Information Supplementary Figures 1-3, Supplementary Notes 1-3 and Supplementary References
