# Supplementary material for: A two-dimensional algebraic quantum liquid produced by an atomic simulator of the quantum Lifshitz model
Source: Nat Commun. 2015 Aug 13;6:8012. doi: 10.1038/ncomms9012 (PMC4557332; doi:10.1038/ncomms9012)
Supplement: Supplementary Information — Supplementary Figures 1-3, Supplementary Notes 1-3 and Supplementary References [file ncomms9012-s1.pdf]

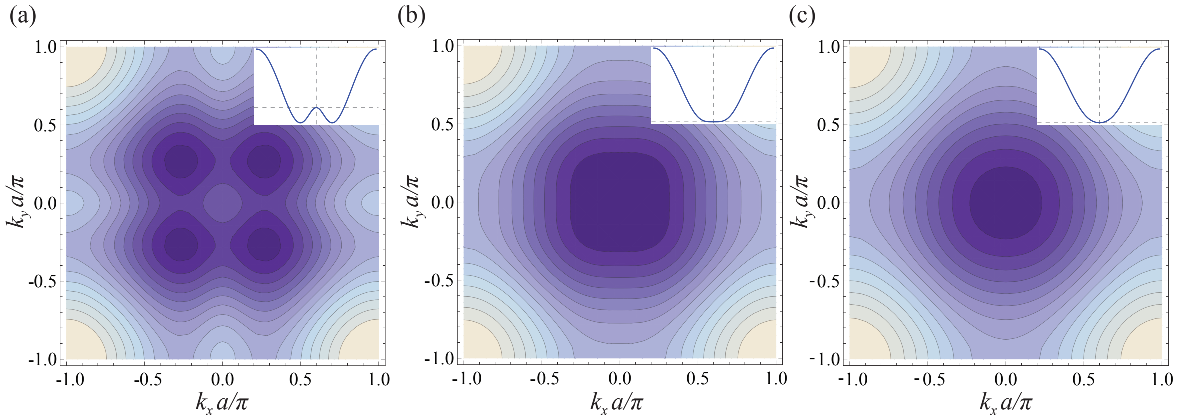

Supplementary Figure 1: Contour plot for typical dispersions at and away from the critical point for the shaken square lattices. Inset: dispersion along $k_x = k_y$. (a) When $\delta^{\mathrm{L}} < \delta_{\mathrm{c}}^{\mathrm{L}+}$, there are four minimum. If one expands the energy around each minimum, the dispersion is also quadratic along both the x and y direction. This is different from the Raman scheme in the continuum, where the rotation symmetry in the momentum space still ensures one quartic dispersion on one side of the critical point. (b) When $\delta^{\mathrm{L}} = \delta_{\mathrm{c}}^{\mathrm{L}+}$, the dispersion becomes quartic. (c) When $\delta^{\mathrm{L}} > \delta_{\mathrm{c}}^{\mathrm{L}+}$, there is a single minimum and the low-energy dispersion is quadratic.

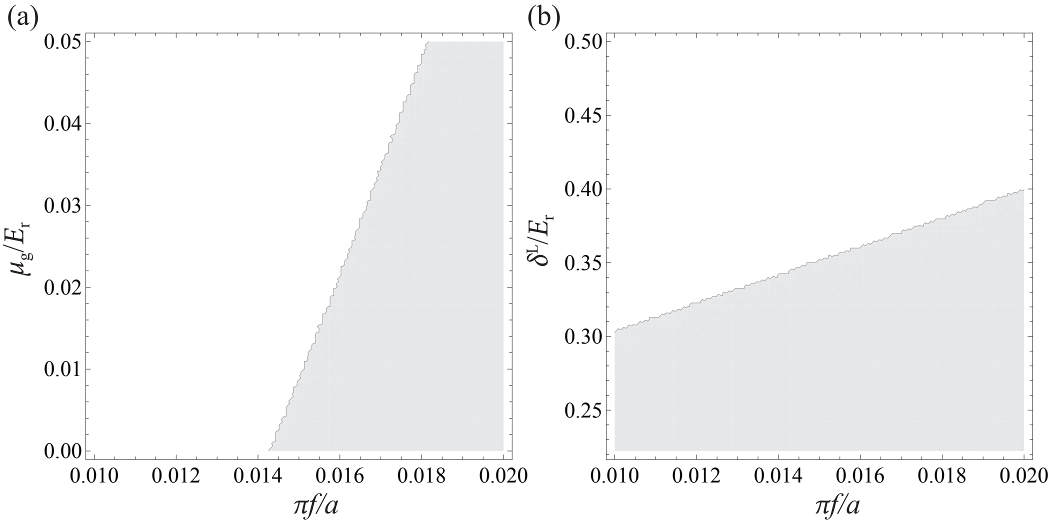

Supplementary Figure 2: Mean field phase diagram for the shaken lattice model. Unshaded and shaded region represent the zero- and finite- momentum phases respectively. (a) Phase diagram on the μ_g-f plane, where $\mu_g = g\rho_0$ characterizes the interaction strength and we fix $\delta^{\mathrm{L}} = 0.36E_r$. As interaction is turned on, the critical shaking amplitude increases. (b) Phase diagram on the δ^{L}-f plane with interaction strength fixed at $\mu_g = 0.02E_r$.

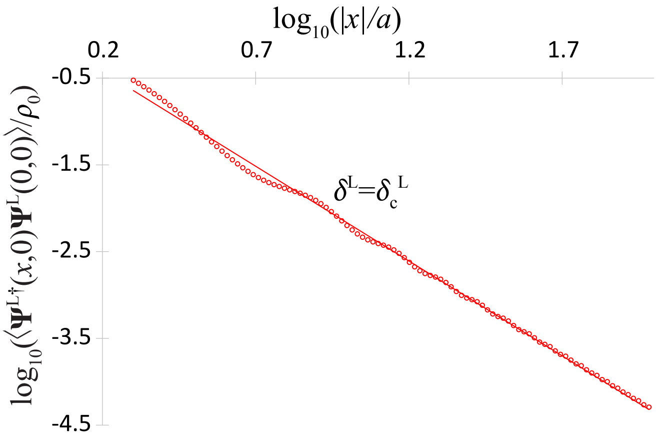

Supplementary Figure 3: Log-log plot for the correlation function in the shaken lattice model at critical point. The correlation function decays algebraically, as seen in the agreement between the data points and the linear fit (solid line).

Supplementary Note 1

Quartic dispersions in shaken lattices

By shaking a lattice, an effective spin-orbit coupling can be produced, where band indices play the role of spin[1,2]. If one shakes a one-dimensional lattice, it has been shown that a quartic dispersion could be produced[1-3]. This method can be straightforwardly generalized to two dimensions.

For a shaken square lattice with the lattice potential $V(x,y) = V_0\left(\cos\left(\frac{2\pi}{a}(x+f\cos\omega t)\right)+\cos\left(\frac{2\pi}{a}(y+f\cos\omega t)\right)\right)$, the Hamiltonian is given in equation (4) of the main text. The interband coupling C can be written as

$$C = -\int dxdy \frac{V_0}{2} J_1\left(\frac{\pi f}{a}\right) W_s(x,y)\sin\left(\frac{2\pi}{a}x\right) W_{p_x}(x,y) = -\int dxdy \frac{V_0}{2} J_1\left(\frac{\pi f}{a}\right) W_s(x,y)\sin\left(\frac{2\pi}{a}y\right) W_{p_y}(x,y) \tag{1}$$

where $J_1(\pi f/a)$ is the Bessel function, and $W_s(x,y)$, $W_{p_x}(x,y)$ and $W_{p_y}(x,y)$ are the Wannier wave functions of the three bands. In the small coupling limit $|C| \ll \delta^{\rm L}$,

$$\beta_0^{\rm L} = \frac{a^4}{24}\left(-t_s - \frac{|C|^2(t_s+t_p)(\delta^{\rm L}+5(t_s+t_p)))}{(t_s+t_p-\delta^{\rm L})^3}\right) \tag{2}$$

A slight difference with the Raman scheme is that the lattice potential reduces the symmetry to a four-fold one, instead of a full rotation symmetry in the momentum space.

The quadratic term vanishes at the critical values $\delta_{\rm c}^{\rm L\pm}$,

$$\delta_{\rm c}^{L\pm} = t_s + t_p \pm \left(\frac{t_s+t_p}{t_s}\right)^{\frac{1}{2}} |C| \tag{3}$$

At $\delta_{\rm c}^{\rm L+}$,

$$\beta_0^{\rm L} = \frac{1}{4}\frac{t_s}{|C|}\sqrt{t_s(t_s+t_p)} > 0 \tag{4}$$

Away from the critical point, however, the dispersion becomes quadratic, as show in Supplementary Fig. 1.

Supplementary Note 2

Effective theory for $0 < \eta \le 1$

We consider the two-dimensional Hamiltonian

$$\mathcal{H} = \int d^2\mathbf{r}\left\{\mathbf{\Psi}^\dagger \frac{1}{2m}\left[\mathbf{p}^2 - 2\lambda(\sigma_x p_x + \eta\sigma_y p_y) + 2\lambda\Omega\sigma^z\right]\mathbf{\Psi}\right\} + \int d^2\mathbf{r}\left\{\left(g_0+\frac{g_s}{2}\right)[\mathbf{\Psi}^\dagger\mathbf{\Psi}]^2 - \frac{g_s}{2}[\mathbf{\Psi}^\dagger\sigma_z\mathbf{\Psi}]^2\right\}, \tag{5}$$

with $0 < \eta \le 1$ characterizing the spatial anisotropy in spin-orbit coupling. The system is analyzed with the ansatz

$$\mathbf{\Psi} = \exp(i\sigma^y\pi/4)\sqrt{\rho}e^{i\theta}\begin{pmatrix} -\sin(\phi/2)\,e^{-i\chi/2} \\ \cos(\phi/2)\,e^{+i\chi/2}\end{pmatrix}. \tag{6}$$

Mean field solution that minimizes the energy functional is found, and it takes different forms for Ω larger than or smaller than its critical value $\Omega_c = \lambda - g_s m\rho_0/\lambda$:

$$\begin{aligned}\theta_0 =& \lambda\cos\phi_0 x;\\ \sin\phi_0 =& \begin{cases}\Omega/\Omega_c & \text{for } \Omega < \Omega_c\\ 1 & \text{for } \Omega \geq \Omega_c\end{cases};\\ \chi_0 =& 0.\end{aligned} \tag{7}$$

The long-wavelength limit of the system is studied by constructing Gaussian effective theories around the mean-field solution through incorporating fluctuations $\rho \to \rho_0 + \delta\rho$, $\phi \to \phi_0 + \delta\phi$, $\chi \to \chi_0 + \delta\chi$ and $\theta \to \theta_0 + \theta$.

1. $\Omega \geq \Omega_c$
   Expanding the Lagrangian about the mean-field solution, up to quadratic order of the fluctuations we have

$$\begin{aligned}\mathcal{L} \approx& \delta\rho\left[g_0 - \frac{1}{8m\rho_0}\nabla^2\right]\delta\rho + \delta\rho\left[i\partial_\tau\theta + \frac{\lambda}{2m}\eta\partial_y\delta\phi - \frac{\lambda}{2m}\partial_x\delta\chi\right] + \delta\phi\left[\frac{\rho_0\lambda^2}{2m}Z_0 - \frac{\rho_0}{8m}\nabla^2\right]\delta\phi\\ &+ \delta\phi\left[-i\frac{\rho_0}{2}\partial_\tau\delta\chi + \frac{\rho_0\lambda}{m}\partial_x\theta\right] + \delta\chi\left[\frac{\rho_0\lambda^2}{2m}Z_0 - \frac{\rho_0}{8m}\nabla^2\right]\delta\chi + \delta\chi\left[\eta\frac{\lambda\rho_0}{m}\partial_y\theta\right] - \frac{\rho_0}{2m}\theta\nabla^2\theta,\end{aligned} \tag{8}$$

   where

$$Z_0 = 1 + \frac{\Omega - \Omega_c}{\lambda} \geq 1 \tag{9}$$

   is defined in such a way that it is dimensionless and equals to 1 at $\Omega = \Omega_c$.

   The fields $\delta\rho$, $\delta\phi$ and $\delta\chi$ are all gapped and can be integrated out, giving the effective Lagrangian for θ

$$\begin{aligned}\mathcal{L}'(\theta) =& \theta\left[-\frac{1}{4g_0}\partial_\tau^2 - \frac{\rho_0(Z_0-1)}{2mZ_0}\partial_x^2 - \frac{\rho_0(Z_0-\eta^2)}{2mZ_0}\partial_y^2 + \frac{\rho_0}{8m\lambda^2 Z_0^2}\partial_x^4 + \frac{(1+\eta^2)\rho_0}{8m\lambda^2 Z_0^2}\partial_x^2\partial_y^2 + \frac{\eta^2\rho_0}{8m\lambda^2 Z_0^2}\partial_y^4\right.\\ &\left.+ \left(\frac{\lambda^2 Z_0 + g_0 m\rho_0}{8g_0\lambda^4 Z_0^3} + \frac{1-Z_0}{32g_0^2 m\rho_0 Z_0}\right)\partial_\tau^2\partial_x^2 + \left(\frac{\eta^2(\lambda^2 Z_0 + g_0 m\rho_0)}{8g_0\lambda^4 Z_0^3} + \frac{\eta^2 - Z_0}{32g_0^2 m\rho_0 Z_0}\right)\partial_\tau^2\partial_y^2\right]\theta.\end{aligned} \tag{10}$$

   In particular, for the isotropic case with $\eta = 1$ we obtain

$$\mathcal{L}(\theta) = \theta\left[-\frac{1}{4g_0}\partial_\tau^2 - \frac{\rho_0(Z_0-1)}{2mZ_0}\nabla^2 + \frac{\rho_0}{8m\lambda^2 Z_0^2}(\nabla^2)^2 + \left(\frac{\lambda^2 Z_0 + g_0 m\rho_0}{8g_0\lambda^4 Z_0^3} + \frac{1-Z_0}{32g_0^2 m\rho_0 Z_0}\right)\partial_\tau^2\nabla^2\right]\theta. \tag{11}$$

   Alternatively, at the critical field $\Omega = \Omega_c$, we have

$$\begin{aligned}\mathcal{L}'(\theta) =& \theta\left[-\frac{1}{4g_0}\partial_\tau^2 - \frac{\rho_0(1-\eta^2)}{2m}\partial_y^2 + \frac{\rho_0}{8m\lambda^2}\partial_x^4 + \frac{(1+\eta^2)\rho_0}{8m\lambda^2}\partial_x^2\partial_y^2 + \frac{\eta^2\rho_0}{8m\lambda^2}\partial_y^4\right.\\ &\left.+ \left(\frac{\lambda^2 + g_0 m\rho_0}{8g_0\lambda^4}\right)\partial_\tau^2\partial_x^2 + \left(\frac{\eta^2(\lambda^2 + g_0 m\rho_0)}{8g_0\lambda^4} + \frac{\eta^2 - 1}{32g_0^2 m\rho_0}\right)\partial_\tau^2\partial_y^2\right]\theta,\end{aligned} \tag{12}$$

   in which the $\sim \partial_x^2$ term is suppressed, leading to the vanishing of T_{BKT}.

   At the quantum critical point $\Omega = \Omega_c$ and $\eta = 1$, we have $Z_0 = 1$ and the usual dominant spatial derivative $\sim \nabla^2$ is suppressed, giving the Quantum Lifshitz model

$$\mathcal{L}_c(\theta) = \theta\left[-\frac{1}{4g_0}\partial_\tau^2 + \frac{\rho_0}{8m\lambda^2}(\nabla^2)^2\right]\theta, \tag{13}$$

   where the term $\sim \partial_\tau^2\nabla^2$ can be dropped as it now enters as a higher order correction in the absence of the usual $\sim \nabla^2$ term.

2. $\Omega < \Omega_c$
   In the same manner the Lagrangian is expanded as

$$\begin{aligned}\mathcal{L} \approx& \delta\rho\left[\tilde{g}_0 - \frac{1}{8m\rho_0}\nabla^2\right]\delta\rho + \delta\rho\left[i\partial_\tau\theta + \frac{i}{2}\cos\phi_0\partial_\tau\delta\chi - (g_s\rho_0\cos\phi_0\sin\phi_0)\delta\phi + \frac{\lambda}{2m}\eta\partial_y\delta\phi - \frac{\lambda}{2m}\sin^2\phi_0\partial_x\delta\chi\right]\\ &+ \delta\phi\left[\left(\frac{\lambda^2\rho_0}{2m} - \frac{g_s\rho_0^2}{2}\cos^2\phi_0\right) - \frac{\rho_0}{8m}\nabla^2\right]\delta\phi + \delta\phi\left[-\frac{i}{2}\rho_0\sin\phi_0\partial_\tau\delta\chi - \frac{\lambda\rho_0}{2m}\sin\phi_0\cos\phi_0\partial_x\delta\chi + \frac{\lambda\rho_0}{m}\sin\phi_0\partial_x\theta\right]\\ &+ \delta\chi\left[\frac{\lambda^2\rho_0}{2m}\sin^2\phi_0 - \frac{\rho_0}{8m}\nabla^2\right]\delta\chi + \delta\chi\left[\frac{\eta\lambda\rho_0}{m}\sin\phi_0\partial_y\theta - \frac{\rho_0}{2m}\cos\phi_0\nabla^2\theta\right]\\ &- \frac{\rho_0}{2m}\theta\nabla^2\theta\end{aligned} \tag{14}$$

where $\tilde{g}_0 = g_0 + g_s \cos^2\phi_0/2$. Note the additional dependence on ϕ_0 as it is now a function of Ω.

In integrating out each of the massive fields $\delta\rho$, $\delta\phi$ and $\delta\chi$, the coefficients in the effective Lagrangian receive corrections and the expressions are substantially more complicated than those for $\Omega \geq \Omega_c$. The corrections are expressed in terms of symbols $Z's$ and $Y's$, which are all defined in such a way that they are dimensionless and equal to 1 at $\Omega = \Omega_c(\eta)$. The effective Lagrangian for θ is

$$\begin{aligned}\mathcal{L}'(\theta) =&\theta\left[-\frac{1}{4g_0}Z^\theta_{\tau\tau}\partial_\tau^2 - \frac{\rho_0}{2m}(1-Z^\theta_{xx})\partial_x^2 - \frac{\rho_0(1-\eta^2)}{2m}\partial_y^2 + i\frac{\rho_0}{2\lambda}(1-Z^\theta_{\tau x})\partial_\tau\partial_x\right]\theta\\ &+\theta\left[\frac{\lambda^2+g_0 m\rho_0}{8g_0\lambda^4}Z^\theta_{\tau\tau xx}\,\partial_\tau^2\partial_x^2 + \left(\frac{\eta^2(\lambda^2+g_0m\rho_0)}{8g_0\lambda^4} + \frac{\eta^2-1}{32g_0^2m\rho_0}\right)Z^\theta_{\tau\tau yy}\partial_\tau^2\partial_y^2\right.\\ &\quad -i\frac{1}{g_0m\lambda}(1-Z^\theta_{\tau xyy})\,\partial_\tau\partial_x\partial_y^2 - i\frac{1}{g_0m\lambda}(1-Z^\theta_{\tau xxx})\,\partial_\tau\partial_x^3\\ &\quad\left.+\frac{\rho_0}{8m\lambda^2}Z^\theta_{xxxx}\partial_x^4 + \frac{(1+\eta^2)\rho_0}{8m\lambda^2}Z^\theta_{xxyy}\partial_x^2\partial_y^2 + \frac{\eta^2\rho_0}{8m\lambda^2}Z^\theta_{yyyy}\partial_y^4\right]\theta,\end{aligned} \tag{15}$$

and the lengthy expressions for Z and Y in terms of the system parameters are listed in the last section of this supplementary note. Note also that the coefficients of the effective Lagrangian are continuous at $\Omega = \Omega_c$.

Condensate fraction and correlation functions

The effective Lagrangian was computed via derivative expansion and terms up to 4th order in derivatives are kept. The most general effective Lagrangian considered can be parameterize as

$$\begin{aligned}\mathcal{L}(\theta) =&\alpha_\tau(\partial_\tau\theta)^2 + \alpha_x(\partial_x\theta)^2 + \alpha_y(\partial_y\theta)^2 + \beta_x(\partial_x^2\theta)^2 + \beta_y(\partial_y^2\theta)^2 + \beta_{xy}(\partial_x\partial_y\theta)^2 +\\ &+ i\gamma_x(\partial_\tau\theta)(\partial_x\theta) + \zeta_x(\partial_\tau\partial_x\theta)^2 + \zeta_y(\partial_\tau\partial_y\theta)^2 + i\epsilon_{xxx}(\partial_\tau\partial_x\theta)(\partial_x^2\theta) + i\epsilon_{xyy}(\partial_\tau\partial_x\theta)(\partial_y^2\theta)\end{aligned} \tag{16}$$

where terms like $\sim(\partial_\tau\theta)(\partial_y\theta)$, which can contribute to the observables to the same order of our calculations, are not generated in integrating out the heavy modes and are therefore absent.

The condensate density, given by $n_0 = n_{0\uparrow} + n_{0\downarrow} = \rho_0 e^{-\langle\theta^2\rangle}$, is found by numerically evaluating the integral

$$\langle\theta^2\rangle = \int\frac{d\omega d^2\mathbf{q}}{(2\pi)^3}\frac{1}{A\omega^2 + iB\omega + C} = \frac{1}{8\pi^2}\int d^2\mathbf{q}\frac{1}{\sqrt{AC + B^2/4}} \tag{17}$$

where

$$\begin{aligned} A =&\alpha_\tau + \zeta_x\, q_x^2 + \zeta_y\, q_y^2\\ B =&\gamma_x q_x + \epsilon_{xyy}q_xq_y^2 + \epsilon_{xxx}q_x^3\\ C =&\alpha_x\, q_x^2 + \alpha_y\, q_y^2 + \beta_x\, q_x^4 + \beta_y\, q_y^4 + \beta_{xy}\, q_x^2q_y^2\\ \Rightarrow AC + B^2/4 \approx& \left(\alpha_\tau\alpha_x + \gamma_x^2/4\right)q_x^2 + (\alpha_\tau\alpha_y)q_y^2 + \left(\alpha_\tau\beta_x + \alpha_x\zeta_x + \gamma_x\epsilon_{xxx}/2\right)q_x^4\\ &+ (\alpha_\tau\beta_y + \alpha_y\zeta_y)q_y^4 + \left(\alpha_\tau\beta_{xy} + \alpha_x\zeta_y + \alpha_y\zeta_x + \gamma_x\epsilon_{xyy}/2\right)q_x^2q_y^2\\ =&\alpha_\tau\left(\tilde{\alpha}_xq_x^2 + \tilde{\alpha}_yq_y^2 + \tilde{\beta}_xq_x^4 + \tilde{\beta}_yq_y^4 + \tilde{\beta}_{xy}q_x^2q_y^2\right)\end{aligned} \tag{18}$$

Similarly, the correlation function $\left\langle\mathbf{\Psi}^\dagger(\mathbf{r})\mathbf{\Psi}(\mathbf{0})\right\rangle = \rho_0 e^{-\langle(\theta(\mathbf{r})-\theta(\mathbf{0}))^2\rangle/2}$ is found by numerically evaluating

$$\left\langle(\theta(\mathbf{r}) - \theta(\mathbf{0}))^2\right\rangle = \frac{1}{8\pi^2}\int d^2\mathbf{q}\frac{1-\cos(\mathbf{q}\cdot\mathbf{r})}{\sqrt{AC + B^2/4}}. \tag{19}$$

The cutoff of the momentum integrals is set by the healing length: $q_{max} = \xi^{-1}$.

For the isotropic case with $\eta = 1$ and $\Omega > \Omega_c$, we have $\tilde{\alpha}_x = \tilde{\alpha}_y = \tilde{\alpha}$ and $\tilde{\beta}_x = \tilde{\beta}_y = \tilde{\beta}_{xy}/2 = \tilde{\beta}$. The characteristic momentum scale is given by $q^* = \sqrt{\tilde{\alpha}/\tilde{\beta}}$ and the integral is given by

$$\left\langle(\theta(\mathbf{r}) - \theta(\mathbf{0}))^2\right\rangle = \frac{1}{4\pi\sqrt{\alpha_\tau\tilde{\beta}}}\int_0^{(q^*\xi)^{-1}} d\tilde{q}\frac{1-J_0(\tilde{q}\tilde{r})}{\sqrt{1+\tilde{q}^2}}, \tag{20}$$

where $\tilde{q}$ and $\tilde{r}$ are both dimensionless (measured in units of $q*$ and $(q^*)^{-1}$). For $|\mathbf{r}| \gg (q^*)^{-1}$, the integral is dominated by the small momentum contribution and so

$$\begin{aligned}\left\langle\mathbf{\Psi}^\dagger(\mathbf{r})\mathbf{\Psi}(\mathbf{0})\right\rangle \approx&\rho_0\exp\left(-\frac{1}{8\pi\sqrt{\alpha_\tau\tilde{\alpha}}}\left(\frac{1}{\xi} - \frac{1}{r}\right)\right)\\ \to&\rho_0\exp\left(-\frac{1}{8\pi\xi\sqrt{\alpha_\tau\tilde{\alpha}}}\right) \text{ as } r\to\infty,\end{aligned} \tag{21}$$

giving the standard long-range correlation in the condensate.

Alternatively, for intermediate values of $|\mathbf{r}|$ such that $\xi \ll |\mathbf{r}| \ll (q^*)^{-1}$, the large momentum contribution of the integral dominates and it gives a power-law correlation function

$$\left\langle \mathbf{\Psi}^\dagger(\mathbf{r})\mathbf{\Psi}(\mathbf{0})\right\rangle \sim \rho_0 \left(\frac{|\mathbf{r}|}{\xi}\right)^{\frac{1}{2K}}, \tag{22}$$

where $K = 4\pi\sqrt{\alpha_\tau\tilde{\beta}}$ is the effective Luttinger liquid parameter. As such the correlation function has a crossover behavior set by the characteristic length scale $(q^*)^{-1}$. In the limit $\Omega \to \Omega_c$, $(q^*)^{-1}$ diverges and therefore the correlation function is dominated by the power-law behavior.

Expressions for Z's and Y's in the effective Lagrangian for $\Omega < \Omega_c$

$$Z_0^\phi = 1 - \frac{m g_s \rho_0 (g_s + 2g_0)}{2\lambda^2 \tilde{g}_0}\cos^2\phi_0; \qquad Z_2^\phi = 1 + \frac{g_s^2 \sin^2\phi_0}{4\tilde{g}_0^2}\cos^2\phi_0;$$

$$Z_{\tau\tau}^\theta = \frac{g_0}{\tilde{g}_0} + \frac{g_s^2 g_0 m \rho_0 \sin^2\phi_0}{2\tilde{g}_0^4\lambda^2 Z_0^\phi}\cos^2\phi_0; \qquad Z_{xx}^\theta = \frac{\sin^2\phi_0}{Z_0^\phi}; \qquad Z_{\tau x}^\theta = 1 - \frac{g_s \sin^2\phi_0}{\tilde{g}_0 Z_0^\phi}\cos\phi_0;$$

$$Z_{\tau\tau xx}^\theta = \frac{(Y_{\tau x}^{\chi\theta})^2(\lambda^2 + 2g_0 m\rho_0)^2 - Y_{\tau\tau xx}^{\theta\theta}\sin^2\phi_0\lambda^4 - 4g_0 m\rho_0\lambda^2(1 - Y_{\tau\tau}^{\chi\theta})(1 - Y_{xx}^{\chi\theta})}{4g_0 m\rho_0(\lambda^2 + g_0 m\rho_0)\sin^2\phi_0};$$

$$Z_{\tau\tau yy}^\theta = \frac{4\eta^2 g_0^2 m^2\rho_0^2 Z_{\tau\tau}^\chi + 4g_0 m\rho_0\lambda^2[(Y_{\tau\tau}^{\chi\theta} - 1)\cos\phi_0 + \eta^2 Y_{\tau\tau y}^{\chi\theta}\sin\phi_0] - Y_{\tau\tau yy}^{\theta\theta}\sin^2\phi_0\lambda^4}{(\eta^2(\lambda^2 + 2g_0 m\rho_0)^2 - \lambda^4)\sin^2\phi_0};$$

$$Z_{\tau xyy}^\theta = 1 - \frac{(\lambda^2 + 2g_0 m\rho_0)Y_{\tau x}^{\chi\theta}\cos\phi_0 + 2\lambda^2[4\eta^2(Z_{\tau x}^\chi - 1) + \eta^2(1 - Y_{\tau xy}^{\chi\theta})\sin\phi_0 + 4(Y_{\tau xyy}^{\theta\theta} - 1)\sin^2\phi_0]}{8\lambda^2\sin^2\phi_0};$$

$$Z_{\tau xxx}^\theta = 1 - \frac{(\lambda^2 + 2g_0 m\rho_0)(1 - Y_{xx}^{\chi\theta})Y_{\tau x}^{\chi\theta} + 8\lambda^2(Y_{\tau xxx}^{\theta\theta} - 1)\sin^2\phi_0}{8\lambda^2\sin^2\phi_0};$$

$$Z_{xxxx}^\theta = Y_{xxxx}^{\theta\theta} - \frac{(Y_{xx}^{\chi\theta} - 1)^2}{\sin^2\phi_0}; \qquad Z_{yyyy}^\theta = \frac{\eta^2 - \cos^2\phi_0}{\eta^2\sin^2\phi_0};$$

$$Z_{xxyy}^\theta = \frac{Y_{xxyy}^{\theta\theta}\sin^2\phi_0 + Z_{xx}^\chi\eta^2}{(1 + \eta^2)\sin^2\phi_0} + \frac{4g_0 m\rho_0(Y_{xx}^{\chi\theta} - 1)\cos\phi_0 - \eta^2\lambda^2(Z_{xx}^\chi + Y_{xxyy}^{\theta\theta}\sin^2\phi_0 - 2Y_{xxy}^{\chi\theta}\sin\phi_0)}{2g_0 m\rho_0(1 + \eta^2)\sin^2\phi_0};$$

$$Z_{\tau\tau}^\chi = \frac{g_0^2\sin^2\phi_0}{\tilde{g}_0^2 Z_0^\phi} + \frac{\lambda^2}{2\tilde{g}_0 m\rho_0}\cos^2\phi_0; \qquad Z_{xx}^\chi = \frac{g_0(2\tilde{g}_0 m\rho_0 - \lambda^2\sin^4\phi_0)}{\tilde{g}_0(2g_0 m\rho_0 - \lambda^2)} - \frac{g_0(g_s + 2g_0)^2 m\rho_0\sin^2\phi_0}{2\tilde{g}_0^2 Z_0^\phi(2g_0 m\rho_0 - \lambda^2)}\cos^2\phi_0;$$

$$Z_{\tau x}^\chi = 1 - \frac{g_0\tilde{g}_0\lambda^2 Z_0^\phi - (g_s + 2g_0)g_0^2 m\rho_0}{8\tilde{g}_0^2\lambda^2 Z_0^\phi}\sin^2\phi_0\cos\phi_0; \qquad Y_{\tau\tau}^{\chi\theta} = 1 - \frac{g_0\tilde{g}_0\lambda^2 Z_0^\phi - g_s g_0^2 m\rho_0\sin^2\phi_0}{\tilde{g}_0^2\lambda^2 Z_0^\phi}\cos\phi_0;$$

$$Y_{xx}^{\chi\theta} = 1 - \frac{2\tilde{g}_0 Z_0^\phi + (g_s + 2g_0)\sin^2\phi_0}{2\tilde{g}_0 Z_0^\phi}\cos\phi_0$$

$$Y_{\tau x}^{\chi\theta} = \frac{g_0(Z_0^\phi\lambda^2 + 2\tilde{g}_0 m\rho_0)\sin^2\phi_0}{\tilde{g}_0(\lambda^2 + 2g_0 m\rho_0)Z_0^\phi} + \frac{g_s^2 g_0 m\rho_0\sin^4\phi_0}{2\tilde{g}_0^2(\lambda^2 + 2g_0 m\rho_0)Z_0^\phi}\cos^2\phi_0$$

$$Y_{\tau\tau y}^{\chi\theta} = \frac{g_0\sin\phi_0}{\tilde{g}_0 Z_0^\phi}; \qquad Y_{\tau xy}^{\chi\theta} = 1 - \frac{g_0}{\tilde{g}_0 Z_0^\phi}\sin\phi_0\cos\phi_0; \qquad Y_{xxy}^{\chi\theta} = \frac{g_0\sin^3\phi_0}{\tilde{g}_0 Z_0^\phi};$$

$$Y_{\tau\tau xx}^{\theta\theta} = \frac{g_0^2}{\tilde{g}_0^2} + \frac{g_s^2 g_0^2 m\rho_0(Z_0^\phi\lambda^2 + \rho_0\tilde{g}_0 m Z_2^\phi)\sin^2\phi_0}{\tilde{g}_0^3\lambda^4(Z_0^\phi)^2}\cos^2\phi_0;$$

$$Y_{\tau\tau yy}^{\theta\theta} = \frac{g_0^2(Z_0^\phi - \eta^2)}{\tilde{g}_0^2 Z_0^\phi} + \frac{g_s^2 g_0^2 m\rho_0(2\lambda^2 Z_0^\phi - \eta^2\lambda^2 + 2\tilde{g}_0 m\rho_0 Z_2^\phi)\sin^2\phi_0}{2\tilde{g}_0^3\lambda^4(Z_0^\phi)^2}\cos^2\phi_0;$$

$$Y_{\tau xyy}^{\theta\theta} = 1 - \frac{g_s g_0(\lambda^2(Z_0^\phi - \eta^2) + 2\tilde{g}_0 m\rho_0 Z_2^\phi)\sin^2\phi_0}{16\tilde{g}_0^2\lambda^2(Z_0^\phi)^2}\cos\phi_0;$$

$$Y_{\tau xxx}^{\theta\theta} = 1 - \frac{g_s g_0(\lambda^2 Z_0^\phi + 2\tilde{g}_0 m\rho_0 Z_2^\phi)\sin^2\phi_0}{16\tilde{g}_0\lambda^2(Z_0^\phi)^2}\cos\phi_0;$$

$$Y_{xxyy}^{\theta\theta} = \frac{g_0(2\tilde{g}_0 m\rho_0 Z_2^\phi - \eta^2\lambda^2)\sin^2\phi_0}{\tilde{g}_0(2g_0 m\rho_0 - \eta^2\lambda^2)(Z_0^\phi)^2}; \qquad Y_{xxxx}^{\theta\theta} = \frac{Z_2^\phi\sin^2\phi_0}{(Z_0^\phi)^2}.$$

Supplementary Note 3 Effective theory for shaken lattices

We analyze the long-wavelength limit of the four-band model described in the main text and Supplementary Note 1, for which the free single-particle Hamiltonian is given by $H_0(\mathbf{k}) - \mu$, with μ denoting the chemical potential. In particular, we focus on the deep lattice limit, for which we can keep only the on-site interaction terms

$$\begin{aligned}\hat{\boldsymbol{U}} = &\sum_{\mathbf{x},\sigma=s,p_x,p_y,d_{xy}} \frac{U_\sigma}{2} b^\dagger_{\mathbf{x}\sigma} b_{\mathbf{x}\sigma}(b^\dagger_{\mathbf{x}\sigma} b_{\mathbf{x}\sigma} - 1) + 4U_{pp}\sum_{\mathbf{x}} b^\dagger_{\mathbf{x}p_x} b_{\mathbf{x}p_x} b^\dagger_{\mathbf{x}p_y} b_{\mathbf{x}p_y} + U_{pp}\sum_{\mathbf{x}}\left(b^\dagger_{\mathbf{x}p_x} b^\dagger_{\mathbf{x}p_x} b_{\mathbf{x}p_y} b_{\mathbf{x}p_y} + h.c.\right) \\ &+ 4U_{sp}\sum_{\mathbf{x}} b^\dagger_{\mathbf{x}s} b_{\mathbf{x}s}\left(b^\dagger_{\mathbf{x}p_x} b_{\mathbf{x}p_x} + b^\dagger_{\mathbf{x}p_y} b_{\mathbf{x}p_y}\right) + 4U_{sd}\sum_{\mathbf{x}} b^\dagger_{\mathbf{x}s} b_{\mathbf{x}s} b^\dagger_{\mathbf{x}d_{xy}} b_{\mathbf{x}d_{xy}} \\ &+ 4U_{pd}\sum_{\mathbf{x}}\left(b^\dagger_{\mathbf{x}p_x} b_{\mathbf{x}p_x} + b^\dagger_{\mathbf{x}p_y} b_{\mathbf{x}p_y}\right) b^\dagger_{\mathbf{x}d_{xy}} b_{\mathbf{x}d_{xy}}.\end{aligned} \tag{23}$$

Note that we have dropped terms like $U_{sp}\sum_{\mathbf{x}} b^\dagger_{\mathbf{x}s} b^\dagger_{\mathbf{x}s} b_{\mathbf{x}p_x} b_{\mathbf{x}p_x} + h.c.$, which vanish under time-averaging. Deep in the superfluid phase, $\psi_\sigma(\mathbf{x}) = \langle b_{\mathbf{x}\sigma}\rangle$ is smooth on lattice scale and we consider the energy functional of the four-component Schrödinger field $\mathbf{\Psi}^{\mathrm{L}}$ that is given by equation (21) of the main text. To simplify expressions, we define

$$\begin{aligned}\bar{\epsilon} = t_p - t_s; \quad \delta\epsilon = -(t_s + t_p) + \delta^{\mathrm{L}}; \quad \epsilon_p = -t_s + t_p; \\ K_{0x} = K_{0y} = K_{1y} = K_{2x} = t_s a^2/2; \quad K_{1x} = K_{2y} = K_{3x} = K_{3y} = -t_p a^2/2.\end{aligned} \tag{24}$$

In addition, we also absorb the phase of C into the definitions of ϕ and ζ and henceforth we use C to denote its magnitude. In these variables, we have

$$\begin{aligned}\mathcal{E} =& \mathcal{E}_{\mathrm{KE}} - \mu\rho + \bar{\epsilon}\rho\cos^2\chi + \delta\epsilon\rho\cos^2\chi\cos(2\nu) + \epsilon_p\rho\sin^2\chi \\ &+ \sqrt{2}C\rho\sin(2\chi)\left[\cos\varphi\cos\xi\left(\cos\nu\cos\phi + \sin\nu\cos\zeta\right) + \sin\varphi\sin\xi\left(\cos\nu\sin\phi - \sin\nu\sin\zeta\right)\right] \\ &+ \frac{U_s a^2}{2}\rho^2\cos^4\chi\cos^4\nu + \frac{U_p a^2}{4}\rho^2\sin^4\chi(1+\sin^2(2\varphi)) + \frac{U_d a^2}{2}\rho^2\cos^4\chi\sin^4\nu \\ &+ U_{sp}a^2\rho^2\sin^2(2\chi)\cos^2\nu + U_{sd}a^2\rho^2\sin^2(2\nu)\cos^4\chi + U_{pd}a^2\rho^2\sin^2(2\chi)\sin^2\nu \\ &+ \frac{U_{pp}a^2}{2}\rho^2\cos^2(2\varphi)\left(2+\cos(4\xi)\right)\sin^4\chi\end{aligned} \tag{25}$$

where $\mathcal{E}_{\mathrm{KE}}$ is the kinetic energy which can be straightforwardly evaluated from equation (4) and (21) of the main text.

The mean-field solution is analyzed by taking the mean-field values of the massive fields $\rho_0, \chi_0, \nu_0, \varphi_0, \phi_0$, ξ_0 and ζ_0 to be spatially homogeneous, and $\theta = \kappa(x+y)$. The mean-field equations are then obtained by requiring $\partial_{F_0}\mathcal{E} = 0$ for F being any of the massive fields or κ. In particular, we have $\phi_0 = \xi_0 = \zeta_0 = 0$ for weak interactions. Note that when $U_{pp}\rho_0 a^2$ dominates over C, there are additional non-trivial solutions with $\xi_0 \to \pm\pi/4$. These solutions correspond to spin-canting resulting from strong repulsive inter-band interaction between bosons in the p_x and p_y bands, but they are unattainable when $2\sqrt{2}U_{pp}\rho_0 a^2 < C\cot\chi_0\csc^2\chi_0$, which is satisfied in the weakly interacting regime we are focusing on.

The phase-boundary between the zero- and finite- momentum phases is determined by the mean field equation

$$\frac{\partial\mathcal{E}}{\partial\kappa} = \kappa\sum_{l=x,y}\left(2(K_{0l}\cos^2\nu_0 + K_{3l}\sin^2\nu_0)\cos^2\chi_0 + (K_{1l} + K_{2l} - (K_{1l} - K_{2l})\sin(2\varphi_0))\sin^2\chi_0\right) = 0 \tag{26}$$

In the zero-momentum phase, $\kappa = 0$ so Eq.(26) is satisfied trivially and due to the symmetry betwene p_x and p_y bands we have $\varphi_0 = 0$. In the finite momentum phase, however, $\kappa \neq 0$ and $(\varphi_0, \nu_0, \chi_0)$ satisfy Eq.(26) non-trivially. The phase boundary is then determined by requiring $(\varphi_0^{\mathrm{c}} = 0, \nu_0^{\mathrm{c}}, \chi_0^{\mathrm{c}})$ to satisfy the two sets of mean-field equations on both sides of the transition.

For simplicity of the analysis, we focus on the zero-momentum phase, which nonetheless is sufficient to capture the physics at the critical point. The Lagrangian $\mathcal{L} = \sum_{\sigma=0}^{2}\left(\psi_\sigma^*\partial_\tau\psi_\sigma\right) + \mathcal{E}$ is expanded around the mean-field solution by incorporating Gaussian fluctuations $(\rho, \chi, \nu, \varphi, \phi, \xi, \zeta, \theta)$ on top of their mean-field values:

The massive fields $(\rho, \chi, \nu, \varphi, \phi, \xi, \zeta)$ are integrated out and we arrive at the effective Lagrangian for θ:

$$\mathcal{L}^{\mathrm{L}} = \alpha_\tau^{\mathrm{L}}\left(\partial_\tau\theta\right)^2 + \alpha^{\mathrm{L}}\left(\nabla\theta\right)^2 + \beta^{\mathrm{L}}\left(\nabla^2\theta\right)^2 + \beta_{xy}^{\mathrm{L}}(\partial_x\partial_y\theta)^2 + \ldots \tag{27}$$

where "…" denotes terms like $\sim(\partial_\tau\partial_x\theta)^2$ that give only minor quantitative corrections to physical observables.

The coefficients for the quadratic and quartic terms are found in terms of the microscopic parameters as

$$\begin{aligned}\alpha^{\rm L} =&\rho_0\left(\tilde K_{0x}+\tilde K_{1x}+\tilde K_{2x}+\tilde K_{3x}\right)\\ \beta^{\rm L} =&\frac{\rho_0}{2}\left(\frac{\tilde K_{3x}^2\cot\nu_0+(\tilde K_{1x}+\tilde K_{2x}+\tilde K_{3x})^2}{\tilde C\cos\nu_0}+\frac{(\tilde K_{1x}-\tilde K_{2x})^2}{\sqrt{2}\tilde C\sin(\nu_0+\pi/4)+8\tilde U_{pp}\rho_0a^2}\right)-\frac{a^2}{12}\alpha^{\rm L}\\ \beta^{\rm L}_{xy} =&-2\rho_0\frac{(\tilde K_{1x}-\tilde K_{1y})^2}{\sqrt{2}\tilde C\sin(\nu_0+\pi/4)+8\tilde U_{pp}\rho_0a^2}+\frac{a^2}{6}\alpha^{\rm L}\end{aligned} \tag{28}$$

where we have introduced the rescaled parameters

$$\tilde K_0=K_0\cos^2\chi_0\cos^2\nu_0;\ \tilde K_{1,2}=K_{1,2}\frac{\sin^2\chi_0}{2};\ \tilde K_3=K_3\cos^2\chi_0\sin^2\nu_0;\ \tilde C=C\frac{\sin\chi_0\cos\chi_0}{\sqrt{2}};\ \tilde U_{pp}=U_{pp}\frac{\sin^4\chi_0}{4}. \tag{29}$$

At the critical point, $\alpha^{\rm L}_{\rm c}=0$ and the long-wavelength behavior of the system is controlled by the quartic terms. In particular, the system is stable at the critical point since

$$(4\beta^{\rm L}+\beta^{\rm L}_{xy})_{\rm c}=\frac{2\rho_0}{\tilde C}\left[\tilde K_{0x}^2\sec\nu_0+\tilde K_{3x}^2\csc\nu_0\right]>0. \tag{30}$$

We have also verified that the excitation spectrum derived from $\mathcal{L}^{\rm L}$ does recover the single-particle energy at the critical point in the non-interacting limit.

Numerical study on the shaken lattice model

To be more concrete, we study the shaken lattice model numerically. The on-site interaction parameters $U_\star$ are given by

$$U_\sigma=2g\int d\mathbf{x}\,W_\sigma(\mathbf{x})^4;\ U_{\sigma\sigma'}=g\int d\mathbf{x}\,W_\sigma(\mathbf{x})^2W_{\sigma'}(\mathbf{x})^2; \tag{31}$$

where $W_\sigma(\mathbf{x})$ denotes the 2D Wannier functions (WFs) for the σ-band, and g is the contact interaction strength of the bosons. Since we are considering separable static lattice potential $V_s(x,y)=V_0\left(\cos(k_rx)+\cos(k_ry)\right)$, the 2D WFs can be factorized into products of 1D ones. In particular, as we are only interested in the deep-lattice limit it is justified to approximate the 1D WFs by normalized Gaussians

$$W_0(x)\approx\frac{1}{\pi^{1/4}(\xi_0a)^{1/2}}\exp\left[-\frac{1}{2}\left(\frac{x}{\xi_0a}\right)^2\right];\quad W_1(x)\approx\frac{\sqrt{2}}{\pi^{1/4}(\xi_1a)^{3/2}}x\exp\left[-\frac{1}{2}\left(\frac{x}{\xi_1a}\right)^2\right]. \tag{32}$$

where $\xi_{0,1}$ are dimensionless measure of the localization of the WFs, and the 2D WFs are given by

$$W_s(x,y)=W_0(x)W_0(y);\quad W_{p_x}(x,y)=W_1(x)W_0(y);\quad W_{p_y}(x,y)=W_0(x)W_1(y);\quad W_{d_{xy}}(x,y)=W_1(x)W_1(y) \tag{33}$$

We consider a lattice with $V_0=16E_r$, for which all microscopic parameters were found numerically. We take $\rho_0=3a^{-2}$ for the numerical calculations and the mean-field phase-diagrams are shown in Supplementary Fig. 2. The correlation function, plotted in Supplementary Fig. 3, was then numerically found for $\mu_g=g\rho_0=0.02E_r$ and $\pi f/a=0.015$. At the critical point with $\delta^{\rm L}_{\rm c}=0.351E_r$, the correlation function decays algebraically, similar to the case in the Raman scheme.

A recent work [4] has also studied the phase diagram of a shaken square lattice. Without a microscopic calculation, the effective Lagrangian provided in Ref. [4] does not include the cross term $(\partial_x\partial_y\theta)^2$. Such a term in general exists in the presence of interaction as it renders the system non-separable. Our theory including only onsite interactions does not lead to the term $|\Phi\partial\Phi|^2$ in Ref. [4], which is irrelevant to our discussions here. Nevertheless, their conclusion that the phase boundary $f_{\rm c}$ increases with increasing interaction agrees with ours.

Supplementary References

[1] Zhang, S.L., & Zhou, Q., Shaping topological properties of the band structures in a shaken optical lattice. *Phys. Rev. A* **90**, 051601(R) (2014).

[2] Zheng, W., & Zhai, H., Floquet Topological States in Shaking Optical Lattices. *Phys. Rev. A* **89**, 061603 (2014).

[3] Parker, C. V., Ha, L. & Chin, C., Direct observation of effective ferromagnetic domains of cold atoms in a shaken optical lattice. *Nature Phys.* **9**, 769-774 (2013).

[4] Miao, C., Liu, B. & Zheng, W. Quantum phase transition of bosons in a shaken optical lattice. *Phys. Rev. A* **91**, 033404 (2015).
